# Risk of major bleeding at different PT-INR ranges in elderly Japanese patients with non-valvular atrial fibrillation receiving warfarin: a nested case-control study

**DOI:** 10.1186/s40780-015-0036-1

**Published:** 2016-01-11

**Authors:** Atsushi Ohgushi, Takayuki Ohtani, Natsumi Nakayama, Shigeo Asai, Yoshiyuki Ishii, Atsuo Namiki, Manabu Akazawa, Hirotoshi Echizen

**Affiliations:** Department of Hospital Pharmacy, Japan Labour Health and Welfare Organization Kanto Rosai Hospital, 1-1 Kiduki-Sumiyoshi, Nakahara, Kawasaki, Kanagawa 211-8510 Japan; Department of Cardiology, Japan Labour Health and Welfare Organization Kanto Rosai Hospital, 1-1 Kiduki-Sumiyoshi, Nakahara, Kawasaki, Kanagawa 211-8510 Japan; Department of Public Health and Epidemiology, Meiji Pharmaceutical University, 2-522-1 Noshio, Kiyose Tokyo, 204-8588 Japan; Department of Pharmacotherapy, Meiji Pharmaceutical University, 2-522-1 Noshio, Kiyose Tokyo, 204-8588 Japan

**Keywords:** Non-valvular atrial fibrillation, Warfarin, PT-INR, Japanese, Elderly patients

## Abstract

**Background:**

Debate continues about the optimal anticoagulation level for elderly Japanese patients with non-valvular atrial fibrillation (NVAF) receiving warfarin. The Japanese Circulation Society guideline has recommended prothrombin time-international normalized ratios (PT-INR) of 1.6 – 2.6 for elderly patients and 2.0 – 3.0 for non-elderly patients, because previous observational studies indicated increased risk of bleeding when the ratio exceeded 2.6. We aimed to reappraise the relationship between PT-INR and the risk of major bleeding in elderly Japanese patients.

**Methods:**

From the electronic medical records, we selected a cohort of elderly (age ≥ 70 years) Japanese patients with NVAF who were prescribed warfarin for the prevention of thromboembolic diseases between November 2010 and March 2014 at Kanto Rosai Hospital. We identified those who developed major bleeding (cases). For each case, we randomly selected two matched controls by adopting a risk-set sampling method defined by calendar date, age, gender, length of warfarin administration, and the prescriber of warfarin. The risk of major bleeding in patients having PT-INR ≤ 1.49, 1.50 – 1.99, 2.00 – 2.49 (the reference), 2.50 – 2.99, and ≥ 3.00 were compared using the conditional logistic regression method. The study protocol was approved by the IRB before the study was begun.

**Results:**

Among the cohort of 806 elderly patients, we identified 32 cases and selected 64 matched controls. The overall incidence of major bleeding was 3.5 per 100 patient-years. The odds ratios (95 % confidence intervals) for the risk of developing major bleeding in patients with PT-INR ≤ 1.49 (*n* = 20), 1.50 – 1.99 (*n* = 32), 2.00 – 2.49 (*n* = 18), 2.50 – 2.99 (*n* = 10), and ≥ 3.00 (*n* = 16) were 1.0 (0.2, 5.9), 0.3 (0.1, 1.9), 1.0 (reference), 1.2 (0.2, 8.4), and 19.8 (2.0, 198.9), respectively, with a significant difference between ≥ 3.00 and reference.

**Conclusions:**

Among elderly Japanese patients with NVAF, PT-INR 2.0 – 3.0 may be associated with a clinically permissible risk of major bleeding while PT-INR ≥ 3.00 a significant risk. Further studies are warranted to determine whether the risk of major bleeding is significantly lower for PT-INR 2.50 – 2.99 than for PT-INR ≥ 3.00.

## Background

Non-valvular atrial fibrillation (NVAF) is the most prevalent arrhythmia in the elderly and poses substantial morbidity and mortality risks because of an increase in cardiogenic thromboembolic complications [1]. Oral anticoagulant therapy has been shown to be effective in reducing the risk of thromboembolic events in patients of all age groups. While many non-vitamin K antagonist oral anticoagulants (NOACs) have become available, their use for elderly patients is still limited because of a paucity of information regarding their safety profiles in the elderly population [2–5]. As a result, warfarin is still most frequently used in these patients. Nevertheless, debate continues regarding the optimal intensity of warfarin therapy in elderly Japanese patients with NVAF. While the prothrombin time-international normalized ratio (PT-INR) range of 2.0 – 3.0 is recommended for Caucasians regardless of age and for non-elderly Japanese patients, the range of 1.6 – 2.6 has been recommended for elderly (age ≥ 70 years) Japanese patients [6–10].

One of the reasons for recommending a lower PT-INR range (1.6 – 2.6) for elderly Japanese patients is that an observational study on approximately 200 Japanese NVAF patients of all ages for the secondary prevention of stroke demonstrated an increased risk of major bleeding for PT-INR ≥ 2.60 [11]. Unfortunately, the study estimated the risk of major bleeding for PT-INR ≥ 2.60 as a whole, and it remains unclear if PT-INR 2.6 – 3.0 would be associated with an increased risk compared to PT-INR 1.6 – 2.6. Recent large cohort studies conducted in Japanese patients with NVAF confirmed that PT-INR 2.0 – 3.0 should be considered the target range for non-elderly Japanese patients, considering the balance between risk of bleeding and anti-thrombotic efficacy [12]. However, there is a relative lack of information regarding the bleeding risk at PT-INR 2.6 – 3.0 in elderly Japanese patients receiving warfarin [13, 14].

A case-control study design would complement prospective randomized or cohort studies in assessing the risk of outcomes with low event rates (such as major bleeding caused by warfarin). Indeed, an optimal PT-INR range of 2.0 – 3.0 for warfarin was first proposed from case-control studies conducted by Hyleck et al. [15, 16] in the early 1990s. With the availability of electronic medical record systems in community hospitals, hospital pharmacists can now conduct case-control studies using real-world clinical data. In the present study, we aimed to assess whether elderly Japanese patients with PT-INR 2.5 – 3.0 have different risk of major bleeding compared to those with PT-INR 2.0 – 2.5 or ≥ 3.0.

## Methods

### Retrieving case patients developing major bleeding

The present study was performed at the Japan Labour Health and Welfare Organization Kanto Rosai Hospital with 610 beds located in an urban area of Tokyo. Patients eligible for the present study were retrieved retrospectively from the electronic medical records between November 2010 and March 2014, utilizing as indices atrial fibrillation in the diagnosis [I48 by International Statistical Classification of Diseases and Related Health Problems, 10th revision (ICD-10)] and age (≥70 years). We also extracted elderly (age ≥ 70 years) patients who were prescribed warfarin using records of the electronic prescribing system. Patient retrieved from both databases were combined. Then those who were prescribed warfarin for clinical indications other than prevention of thromboembolic events associated with NVAF were excluded to obtain the final cohort for the present case-control study.

The electronic medical records were searched for occurrence of major bleeding events in the cohort using the following ICD-10 codes as event identifiers: D69 (purpura and other hemorrhagic conditions), I60 to 62 (subarachnoid hemorrhage, intracerebral hemorrhage, and other nontraumatic intracranial hemorrhage), K25.0, .2, .4, and .6 (gastric ulcer: acute with hemorrhage or perforation, chronic with hemorrhage or perforation), K26.0, .2, .4, and .6 (duodenal ulcer: acute with hemorrhage or perforation, chronic with hemorrhage or perforation), K27.0, .2, .4, and .6 (peptic ulcer, site unspecified: acute with hemorrhage or perforation, chronic with hemorrhage or perforation), K28.0, .2, .4, and .6 (gastrojejunal ulcer: acute with hemorrhage or perforation, chronic with hemorrhage or perforation), K29.0 (acute hemorrhagic gastritis), K62.5 (hemorrhage of anus and rectum), K92.0, .1, and .2 (hematemesis, melena, gastrointestinal hemorrhage unspecified), and R04 (hemorrhage from respiratory passages). Because no consensus has been reached regarding the criteria for major bleeding, we adopted the following composite criteria by modifying that used in the RE-LY study [17]. Specifically, we considered the recorded bleeding events to be compatible with major bleeding when at least one of the following conditions was met: a reduction of blood hemoglobin level of 2 g/dL or greater from the nearest preceding value, a reduction of hemoglobin level below 8 g/dL, blood transfusion was undertaken, or bleeding events were judged to necessitate hospitalization for transfusion and other necessary therapies. Because the last criterion is less objective and may be inconsistent between attending physicians, we performed an additional analysis by excluding the cases that were considered to develop major bleeding solely based on the last criterion. Whether the cases retrieved from the databases met the criteria of major bleeding was determined by two authors independently: A.O. (pharmacist) and A.N. (physician). Data of PT-INR associated with major bleeding episodes were collected from the medical records when patients visited the outpatient clinic or emergency department. In the case of emergency, the data immediately after admission were collected.

### Assignment of controls

For each case patient, we randomly assigned two control patients by adopting a risk-set sampling method defined by the calendar date of the bleeding event, age, gender, length of warfarin administration, and the prescriber of warfarin [18]. In control patients, data of PT-INR nearest to the calendar date when their corresponding case patients developed major bleeding were used.

### Retrieving clinical data relevant to risk analysis

Age, gender, height, weight, estimated glomerular filtration rate (eGFR), PT-INR, warfarin doses, and other clinical data required to calculate CHADS_2_ and HAS-BLED scores were extracted from the medical records of the retrieved patients [19, 20]. For calculating the HAS-BLED scores we searched for anti-platelet drugs and NSAIDs. In addition, we searched for the concomitant medications which might have inhibited the metabolic activity of CYP2C9 or augmented the anticoagulation effects of warfarin. They included acetaminophen, allopurinol, amiodarone, azole antifungal drugs, cimetidine, fluoroquinolones, macrolide antibiotics, metronidazole, propafenone, SSRIs, statins, sulfa antibiotics. Labile PT-INR [percent time within the therapeutic range in total therapeutic period (TTR) < 60 %] was calculated using the Rosendaal method [21]. The method assumes that changes between consecutive PT-INR measurements are linear over time. We calculated TTR assuming that PT-INR 1.6 – 2.6 is the therapeutic range for elderly Japanese patients with NVAF according to the 2013 guideline of the Japanese Circulation Society [10]. We excluded the following periods from calculation of TTR: within 7 days after the commencement of warfarin therapy and periods when warfarin was discontinued for any reason. We calculated TTR only when the PT-INR data for more than 6 consecutive months were available and when the longest measurement intervals were less than 3 months apart.

### Ethical issues

The present study was planned and conducted in compliance with the Strengthening the Reporting of Observational Studies in Epidemiology (STROBE) statement and the Declaration of Helsinki. The protocol was approved by the institutional review board of Kanto Rosai Hospital (the approval # 2014–14).

### Statistical analysis

Comparisons of continuous variables were performed by paired t-test and those for categorical variables by McNemar’s test. We stratified the case and control patients into five categories according to their PT-INR values (≤1.49, 1.50 – 1.99, 2.00 – 2.49, 2.50 – 2.99, and ≥ 3.00). The risk of major bleeding for each group was evaluated by conditional logistic regression analysis and expressed as odd ratio (OR) and 95 % confidence interval (CI) versus patients with PT-INR 2.00 – 2.49 (reference).

We performed power calculation for the statistical analysis of a case-control study before we began the study, according to the method reported previously [22]. We made the assumption that approximately 10 % of control and 30 % of case patients had been exposed to PT-INR ≥ 2.60. Based on this assumption and a case-control matching ratio of 1:2, we estimated that at least 34 cases and 68 controls are required for detecting a significant difference in the risk (OR) of developing major bleeding with an α error of 5 % and a power of 80 %. In addition, we performed power calculation for a cohort study according to the method reported elsewhere [22]. We assumed that the event rates of major bleeding in patients exposed to PT-INR ≥ 2.60 and < 2.60 were 15 % and 3 % person-years, respectively, according to the data reported in previous study [14] and that the size ratio between the former and latter group was 1:9. A p value less than 0.05 was considered statistically significant throughout the study. The analysis was performed using JMP Pro v.11 software (SAS Institute Inc., Cary, NC, USA).

## Results

**Fig. 1:**
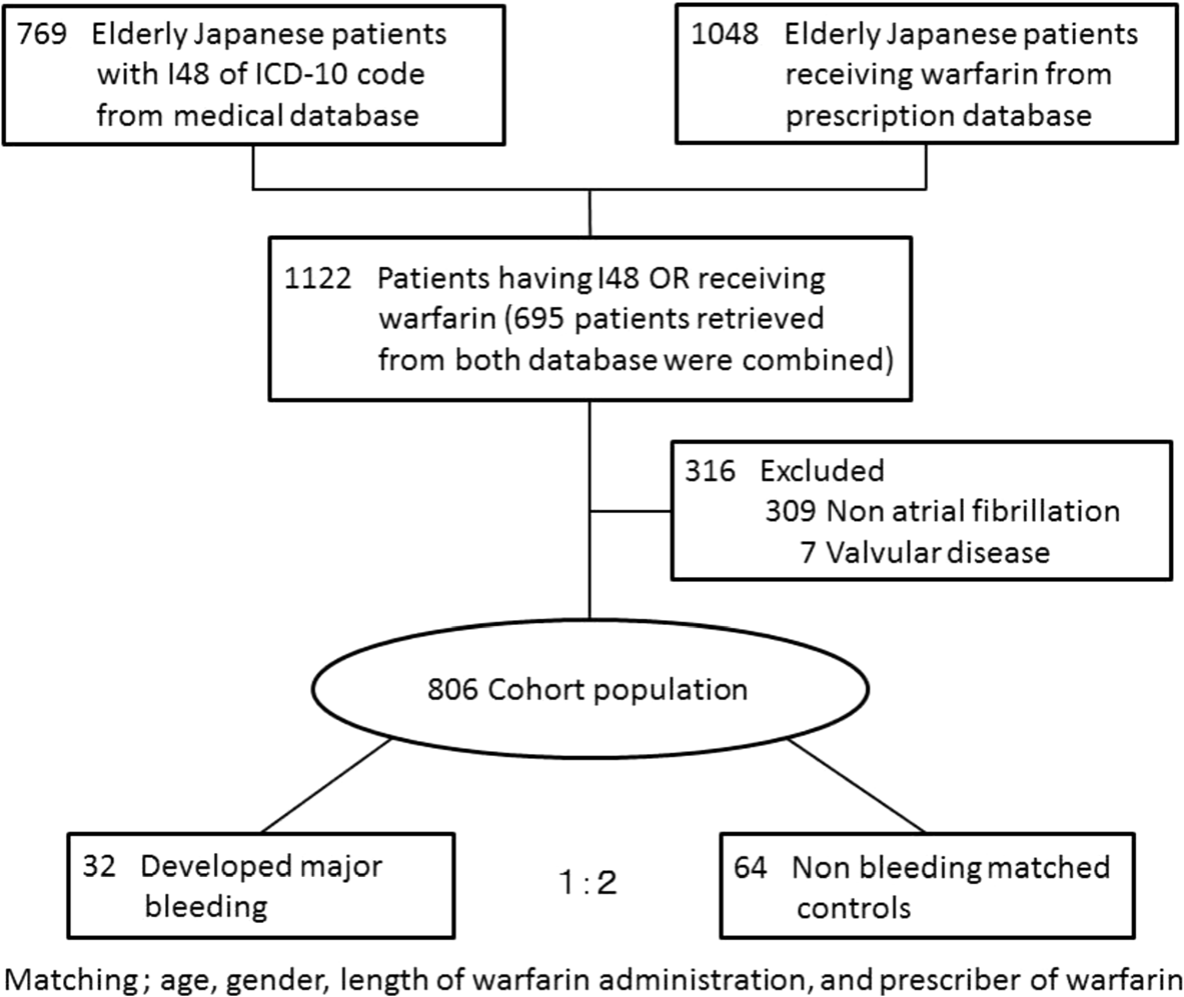
Flowchart of the retrieval, collation and integration of patient data

**Fig. 2:**
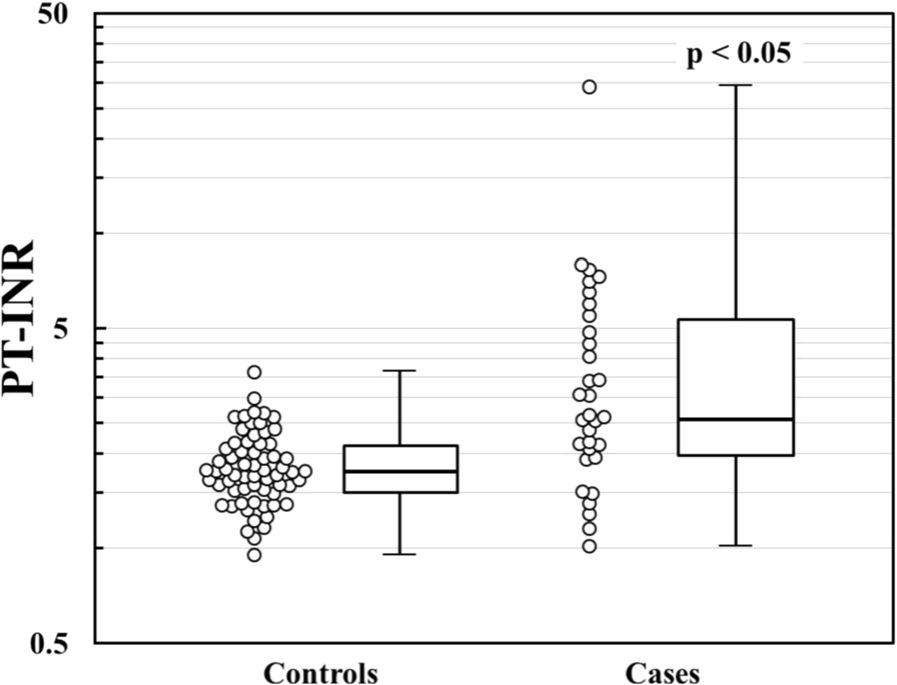
Distribution of prothrombin time-international normalized ratio (PT-INR) in control and case patients. Median PT-INR (interquartile range) for case and control patients are 2.62 (1.97 – 5.30) and 1.75 (1.52 – 2.14), respectively. A significant difference is detected by paired t-test

Seven hundred and sixty-nine elderly patients who were diagnosed with atrial fibrillation were extracted from the electronic medical records. In addition, 1,048 elderly patients prescribed warfarin were extracted from the prescription record database. Combining the two sets of data and collating overlapping patients, we identified 1,122 elderly patients who had atrial fibrillation and/or had received warfarin. Excluding 316 patients who were prescribed warfarin for clinical indications other than atrial fibrillation or who had valvular heart disease, the final study cohort comprised 806 elderly Japanese patients with NVAF who received warfarin (Fig. 1). Among a total of 918 person-years on warfarin therapy in the cohort, we identified 32 major bleeding events (3.5 per 100 patient-years) including 16 gastrointestinal hemorrhages, 8 intracranial hemorrhages, 5 coagulation disorders, and 3 respiratory tract hemorrhages. The number of case patients largely met the estimated size by power calculation. The demographic and clinical characteristics of the case and control patients are listed in Table 1. No significant differences in CHADS_2_ and HAS-BLED scores were observed between the two groups. The CHADS_2_ and HAS-BLED scores (both, 3 ± 1) were identical in the two groups. In addition, there was no significant difference in the numbers of patients receiving CYP2C9 inhibitors or those augment anticoagulation effect of warfarin: 17 of 32 (53 %) cases and 35 of 64 (55 %) controls ingested at least one of the above drugs by McNemar’s test. The PT-INR (median; interquartile range) for the cases (2.62; 1.97 – 5.30) was significantly (*p* < 0.05) greater than the ratio for the controls (1.75; 1.52 – 2.14) (Fig. 2). While approximately 50 % of the case patients showed PT-INR ≥ 2.60, only approximately 10 % of the control patients showed PT-INR ≥ 2.60 (OR 11.8; 95 % CI, 3.8 – 37.1). Collectively, these data largely agree with the preconditions for power calculation.

**Table 1:** Clinical characteristics of case and control patients

Characteristics	Cases (*n* = 32)	Controls (*n* = 64)	*P* value
Age (yr), mean ± SD	81 ± 5	81 ± 5	NS
Gender, M/F	14/18	28/36	NS
PT-INR			
Median PT-INR(Q1,Q3)	2.62 (1.97, 5.30)	1.75 (1.52, 2.14)	<0.05
≤1.49, n (%)	5 (16)	15 (24)	NS
1.50 to 1.99, *n* (%)	3 (9)	29 (45)	<0.05
2.00 to 2.49, *n* (%)	5 (16)	13 (20)	NS
2.50 to 2.99, *n* (%)	4 (12)	6 (9)	NS
≥3.00, *n* (%)	15 (47)	1 (2)	<0.05
eGFR (mL/min/1.73 m^2^), mean ± SD	43 ± 27	49 ± 15	NS
CHADS_2_ score, mean ± SD	3 ± 1	3 ± 1	NS
Congestive heart failure, *n* (%)	19 (59)	19 (30)	<0.05
Hypertension, *n* (%)	22 (69)	50 (78)	NS
Diabetes, *n* (%)	5 (16)	21 (33)	NS
Stroke/TIA, *n* (%)	16 (50)	23 (36)	NS
HAS-BLED score, mean ± SD	3 ± 1	3 ± 1	NS
Impaired renal function, *n* (%)	7 (22)	3 (5)	<0.05
Impaired liver function, *n* (%)	4 (13)	3 (5)	NS
Stroke, *n* (%)	14 (44)	22 (34)	NS
History of bleeding, *n* (%)	10 (31)	14 (22)	NS
Labile PT-INR, *n* (%)	2 (6)	19 (30)	NS
Antiplatelet or NSAID, *n* (%)	12 (38)	24 (38)	NS
Alcohol consumption, *n* (%)	1 (3)	4 (6)	NS

Q1 and 3 represent the upper limits of the first and third interquartile ranges. *TIA* transient ischemic attack, *PT-INR* prothrombin time-international normalized ratio, *eGFR* estimated glomerular filtration rate, *NSAID* non-steroidal anti-inflammatory drug

**Fig. 3:**
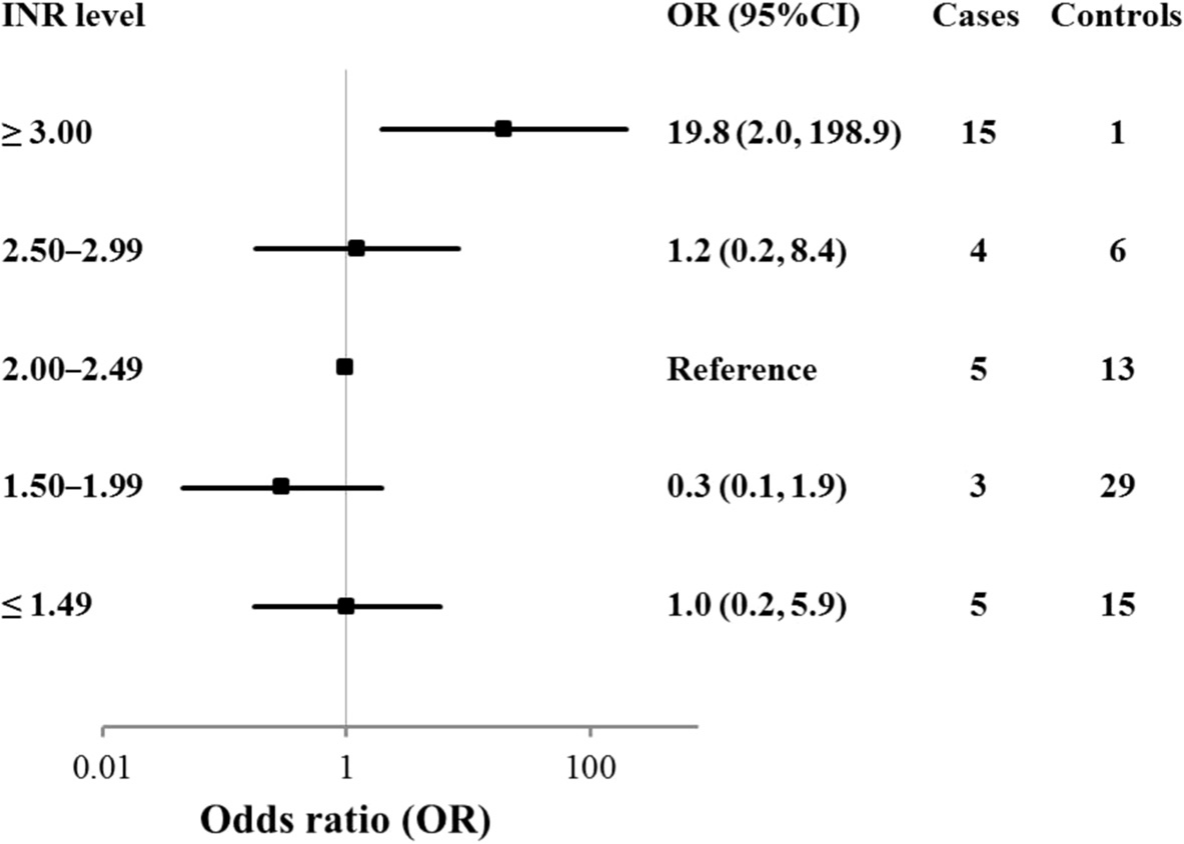
Comparisons of odds ratios (ORs) and 95 % confidence intervals for developing major bleeding stratified by prothrombin time-international normalized ratio (PT-INR). The horizontal bars indicate 95 % confidence intervals (CIs) calculated by the conditional logistic regression method

When the risk of developing major bleeding in patients with different PT-INR ranges were compared using the risk for PT-INR 2.00 – 2.49 as reference (OR = 1), ORs for patients with PT-INRs 2.50 – 2.99, 1.50 – 1.99, and ≤ 1.49 were not significantly different from the reference, whereas OR for patients with PT-INR ≥ 3.00 was significantly (*p* < 0.05) greater than that of the reference (Fig. 3).

In addition, the result of power calculation for a cohort study revealed that one-year follow-up of at least 400 patients (16 patients expected to develop major bleeding) is required to obtain sufficient power for detecting a significant difference in the risk of bleeding between patients exposed to PT-INR ≥ 2.60 and those exposed to < 2.60.

Five patients were considered to develop major bleeding solely by the criterion of clinical judgement of the necessity of admission. When the statistical analysis was performed by excluding these data, essentially similar results were obtained (data are not shown).

## Discussion

To the best of our knowledge, this is the first nested case-control study carried out to assess the relationship between PT-INR and the risk of developing major hemorrhage in elderly Japanese patients with NVAF receiving warfarin. Our data show that patients having PT-INR ≥ 3.00 had approximately 20 times greater risk of developing major bleeding compared to patients having PT-INR 2.00 – 2.49 (reference), whereas those with PT-INR 2.50 – 2.99 appeared to have similar risk (OR 1.23) compared to the reference. Our data support the notion that the upper limit of target PT-INR for elderly Japanese patients with NVAF may be extended to 3.00. Target PT-INR of 2.0 – 3.0 has been recommended for non-elderly Japanese patients. It is noteworthy that both case and control patients participating in the present study had a high risk of thromboembolic complications, because the mean age of both groups was 81 years and both had a median CHADS_2_ score of 3 [19]. In addition, they had a high risk of bleeding during anticoagulation therapy because of their ages and HAS-BLED scores (Table 1). In this context, they represent elderly patients in the real-world and therefore the results of the present study would be relevant to daily medical practice.

Our data in general agree with previous studies. A subgroup analysis of elderly (age ≥ 70 years) patients in the J-Rhythm registry showed that the hazard ratio (95 % CI) of major bleeding in patients with PT-INR 2.00 – 2.59, 2.60 – 2.99, and ≥ 3.00 increased gradually to 2.87 (1.12 – 7.35), 3.99 (1.33 – 11.89), and 7.02 (2.23 – 22.13), respectively, against those who did not receive warfarin. Nevertheless, the 95 % CIs of these groups overlapped substantially [12]. Naganuma et al. conducted a cohort study of 845 elderly Japanese patients with NVAF and found that those with PT-INR ≥ 3.00 had remarkably higher incidence of major bleeding (20 per patient-year) compared to those with PT-INR 2.00 – 2.49 (1.5 per patient-year) and 2.50 – 2.99 (3.4 per patient-year) [14]. However, the 95 % CI for PT-INR 2.50 – 2.99 (95 % CI, 0.9 – 8.5) overlapped substantially with that for 2.00 – 2.49 (95 % CI, 0.6 – 3.2). Collectively, we speculate that it would be practically impossible to obtain statistically significant difference in major bleeding risk between PT-INR 1.6 – 2.6 and 2.6 – 3.0. In contrast, it may be possible to detect a significant difference in bleeding risk between the target PT-INR (irrespective of 1.6 – 2.6 or 2.0 – 3.0) and PT-INR higher than 3.0. To achieve such purpose, a case-control study design is useful, and was used by Hylek et al. [15, 16] to establish the current target PT-INR 2.0 – 3.0 for Caucasians. We calculated that the present study comprising 96 patients had comparable statistical power as a cohort study of 400 patients. Thus a case-control study would be more practicable than a cohort study to estimate the risk of drugs in a special population such as the elderly.

The present study has several drawbacks. First, we were unable to analyze the risk of intracranial hemorrhage at different PT-INR levels separately, because only a small number of events (*n* = 8) were observed in our cohort. A recent study has reported that the risk of intracranial hemorrhage in elderly Japanese may increase approximately four-fold (OR 4.2; 95 % CI, 1.8 – 9.8) at PT-INR 2.5 – 3.0 [13]. Ultimately, the optimal target PT-INR for elderly patients with NVAF should be estimated in light of all-cause mortality as reported by Oden and Fahlen [23]. They performed a medical record linkage study in 42,451 Caucasian patients and found that patients with PT-INR 2.0 – 2.4, 2.5 – 2.9, and 3.0 – 3.4 had all-cause mortalities of 42.3, 47.4, and 67.9 per 1,000 patient-years, respectively. At present, no such data are available for the Japanese population. Second, the present study has insufficient numbers of cases for estimating OR with narrow 95 % CI ranges in patients with the PT-INR ≥ 3.0, primarily because the study was conducted in a single medical center. Third, we did not examine the genetic polymorphisms of VKORC1 and CYP2C9 which are known to influence on the inter-individual variability and risk of bleeding in patients receiving warfarin. As a result, we cannot make any inference on the contribution of these factors to the risk of bleeding. Finally, we cannot analyze the relationship between PT-INR and thrombotic events for warfarin in the present study. For such a purpose we needed to collect cases developing thrombotic events and matched controls.

## Conclusion

The present study demonstrates that PT-INR 2.0 – 3.0 may be associated with a clinically permissible risk of major bleeding while PT-INR ≥ 3.00 a significant risk in elderly Japanese patients with NVAF. The present study warrants further case-control studies with a greater number of patients to obtain conclusive evidence.
